# Persistence landscapes: Charting a path to unbiased radiological interpretation

**DOI:** 10.18632/oncotarget.28671

**Published:** 2024-11-12

**Authors:** Yashbir Singh, Colleen Farrelly, Quincy A. Hathaway, Gunnar Carlsson

**Keywords:** persistence landscape, topology, topological features, radiology

## Abstract

Persistence landscapes, a sophisticated tool from topological data analysis, offer a promising approach to address biases in radiological interpretation and AI model development. By transforming complex topological features into statistically analyzable functions, they enable robust comparisons between populations and datasets. Persistence landscapes excel in noise filtration, fusion bias mitigation, and enhancing machine learning models. Despite challenges in computation and integration, they show potential to improve the accuracy and equity of radiological analysis, particularly in multi-modal imaging and AI-assisted interpretation.

## INTRODUCTION

In the ever-evolving field of medical imaging, the pursuit of unbiased and accurate interpretation remains a paramount challenge. As we continue to leverage advanced technologies and artificial intelligence (AI) in radiology, a novel approach from topological data analysis (TDA) has emerged as a promising tool: persistence landscapes. This sophisticated statistical method for summarizing topological features offers new avenues for addressing biases and enhancing the reliability of radiological interpretations.

### Understanding persistence landscapes

Persistence landscapes, introduced by Bubenik in 2015 [1], are a statistical tool derived from persistence images, which are fundamental in TDA. While persistence images capture the birth and death of topological features across different scales, persistence landscapes transform this information into a sequence of real-valued functions. This transformation preserves the topological information while providing a format more amenable to statistical analysis [2]. The key advantage of persistence landscapes lies in their ability to represent complex topological information in a way that allows for the application of traditional statistical methods. This property makes them particularly valuable in medical imaging, where we often need to compare different populations or datasets to identify patterns or biases [3].

### Comparing populations and datasets in radiology

One of the most promising applications of persistence landscapes in radiology is their potential to reveal demographic or equipment-related biases. By generating persistence landscapes for different subsets of radiological data – such as images from different demographic groups or acquired with different equipment – we can perform statistical comparisons that may uncover subtle but significant differences [4]. For instance, persistence landscapes could be used to compare the topological features of brain MRI scans across different age groups or ethnicities. Any systematic differences in these landscapes might indicate potential biases in image acquisition or interpretation that need to be addressed [5].

### Filtration of noise

One of the inherent strengths of persistence landscapes is their ability to filter out noise while preserving meaningful topological features. This property is particularly valuable in radiological imaging, where image artifacts and noise can significantly impact interpretation [6]. The multi-scale nature of persistence landscapes allows them to capture features that persist across different scales, effectively distinguishing between genuine anatomical structures and transient noise. This filtration effect can lead to more reliable and less biased interpretations of complex imaging data [7].

### Fusion bias mitigation

In the era of multi-modal imaging, where we often combine data from different imaging modalities (e.g., PET/CT, PET/MRI), persistence landscapes offer a unique approach to mitigate fusion bias. By generating persistence landscapes for each modality separately and then comparing or combining them, we can identify discrepancies or biases that might arise from the fusion process [8]. This approach could provide superior integration of multiple imaging modalities, enhancing diagnostic accuracy without introducing new biases or artifacts. It provides a topological perspective on data fusion that complements traditional image registration and fusion techniques [9].

### Statistical soundness of comparison tests

One of the key advantages of persistence landscapes is the statistical soundness they bring to comparison tests. Unlike some other topological summaries, persistence landscapes live in a vector space, allowing for the application of a wide range of statistical tests and machine learning techniques [10]. This property enables rigorous statistical comparisons between different groups of images or between human and AI interpretations. For example, we can use standard statistical tests to determine if the differences in persistence landscapes between two populations are statistically significant, providing a solid foundation for identifying potential biases [11].

### Avoiding machine learning biases

As we increasingly rely on machine learning (ML) in radiological interpretation, persistence landscapes offer a way to mitigate some common ML biases:

1. Cycling Behavior in Boosting: Boosting algorithms can sometimes exhibit cycling behavior, repeatedly misclassifying the same examples. Persistence landscapes, by providing a stable topological summary of the data, can help identify and mitigate this issue by offering a consistent representation of the underlying structure [12].
2. Smoothing in Deep Learning: Deep learning models often struggle with preserving fine details due to their smoothing effect. Persistence landscapes, by capturing multi-scale topological features, can complement deep learning approaches and help preserve important structural details that might otherwise be lost [13].
3. Sampling Bias in Decision Trees: Decision trees are susceptible to sampling bias, potentially leading to overfitting. Persistence landscapes can provide a more robust representation of the data’s topology, helping to guide the tree-building process and reduce the impact of sampling artifacts [14].

By incorporating persistence landscapes into ML pipelines, we can develop more robust and unbiased models for radiological interpretation. This topological perspective can complement traditional feature engineering approaches and help uncover subtle structural patterns that might be missed by conventional ML techniques [15].

### Challenges and future directions

While persistence landscapes offer significant promise in addressing biases in radiological interpretation, several challenges remain:

1. Computational Complexity: Generating persistence landscapes for large-scale radiological datasets can be computationally intensive. Developing more efficient algorithms and leveraging high-performance computing resources will be crucial for widespread adoption [16].
2. Interpretability: While persistence landscapes provide a statistically sound representation of topological features, interpreting these landscapes in the context of specific radiological findings can be challenging. Developing intuitive visualization tools and training programs will be essential to bridge this gap [17].
3. Integration with Existing Workflows: Incorporating persistence landscape analysis into established radiological workflows will require careful planning and validation. Demonstrating the added value of this approach in real-world clinical settings will be crucial for adoption [18].

## CONCLUSIONS

Persistence landscapes represent a powerful new tool in our ongoing efforts to achieve unbiased and accurate radiological interpretation. By providing a statistically sound method for summarizing and comparing topological features in medical images, they offer unique insights that can complement existing analytical approaches. As we continue to refine and validate this technique, persistence landscapes have the potential to play a crucial role in identifying and mitigating biases in radiological practice, whether these biases stem from demographic factors, equipment variations, or the limitations of AI algorithms. The path to truly unbiased radiological interpretation is complex, but with innovative approaches like persistence landscapes, we are equipping ourselves with sophisticated tools to navigate this challenge.
